# Mental illness: a political perspective

**DOI:** 10.1186/1753-6561-7-S5-O20

**Published:** 2013-08-30

**Authors:** Ignazio Cassis

**Affiliations:** 1 National Council, Switzerland

## 

I Cassis, a member of the Swiss National council presented the political perspective regarding mental illnesses. What is the role of drug companies and new definitions in creating a scenario wherein majority of the population are mentally ill? We do not know for sure and whether epidemiology can help in the matter. The responsibility of providing solutions is another vague area. We are not sure whether we improved with the current strategy. These may be the result of a lack of clear institutional framework, lack of financial need assessment and a lack of a common culture in mental health.

